# Early cerebellar deficits in mitochondrial biogenesis and respiratory chain complexes in the KIKO mouse model of Friedreich ataxia

**DOI:** 10.1242/dmm.030502

**Published:** 2017-11-01

**Authors:** Hong Lin, Jordi Magrane, Amy Rattelle, Anna Stepanova, Alexander Galkin, Elisia M. Clark, Yi Na Dong, Sarah M. Halawani, David R. Lynch

**Affiliations:** 1 Departments of Pediatrics and Neurology, Children's Hospital of Philadelphia, Philadelphia, PA 19104, USA; 2 Feil Family Brain and Mind Research Institute, Weill Cornell Medical College, New York, NY 10065, USA; 3 Queen's University Belfast, School of Biological Sciences, Medical Biology Centre, 97 Lisburn Road, Belfast BT9 7BL, UK; 4 Perelman School of Medicine, University of Pennsylvania, Philadelphia, PA 19104, USA

**Keywords:** Cerebellum, Friedreich ataxia, Mitochondrial biogenesis, Respiratory chain complex, Neurodegenerative diseases

## Abstract

Friedreich ataxia (FRDA), the most common recessive inherited ataxia, results from deficiency of frataxin, a small mitochondrial protein crucial for iron-sulphur cluster formation and ATP production. Frataxin deficiency is associated with mitochondrial dysfunction in FRDA patients and animal models; however, early mitochondrial pathology in FRDA cerebellum remains elusive. Using frataxin knock-in/knockout (KIKO) mice and KIKO mice carrying the mitoDendra transgene, we show early cerebellar deficits in mitochondrial biogenesis and respiratory chain complexes in this FRDA model. At asymptomatic stages, the levels of PGC-1α (PPARGC1A), the mitochondrial biogenesis master regulator, are significantly decreased in cerebellar homogenates of KIKO mice compared with age-matched controls. Similarly, the levels of the PGC-1α downstream effectors, NRF1 and Tfam, are significantly decreased, suggesting early impaired cerebellar mitochondrial biogenesis pathways. Early mitochondrial deficiency is further supported by significant reduction of the mitochondrial markers GRP75 (HSPA9) and mitofusin-1 in the cerebellar cortex. Moreover, the numbers of Dendra-labeled mitochondria are significantly decreased in cerebellar cortex, confirming asymptomatic cerebellar mitochondrial biogenesis deficits. Functionally, complex I and II enzyme activities are significantly reduced in isolated mitochondria and tissue homogenates from asymptomatic KIKO cerebella. Structurally, levels of the complex I core subunit NUDFB8 and complex II subunits SDHA and SDHB are significantly lower than those in age-matched controls. These results demonstrate complex I and II deficiency in KIKO cerebellum, consistent with defects identified in FRDA patient tissues. Thus, our findings identify early cerebellar mitochondrial biogenesis deficits as a potential mediator of cerebellar dysfunction and ataxia, thereby providing a potential therapeutic target for early intervention of FRDA.

## Abstract (precis)

**Summary:** Our findings identify early cerebellar mitochondrial biogenesis deficits as a potential mediator of cerebellar dysfunction and ataxia, and indicate a potential therapeutic target for early intervention of Friedreich ataxia.

## INTRODUCTION

Friedreich ataxia (FRDA), the most common autosomal recessive hereditary ataxia, is majorly caused by homozygous expanded guanine-adenine-adenine (GAA) repeats in intron 1 of the frataxin (*FXN*) gene (Campuzano et al., 1996; Lynch et al., 2012; Lynch and Seyer, 2014). This expansion results in chromatin condensation and reduced expression of frataxin (Bidichandani et al., 1998; Campuzano et al., 1997; Chutake et al., 2014, 2015; Grabczyk and Usdin, 2000; Li et al., 2015). Frataxin is a highly conserved mitochondrial protein crucial for iron-sulphur (FeS) cluster formation and ATP production (Bulteau et al., 2004; Fox et al., 2015; Isaya et al., 2004; Li et al., 2015; Lu and Cortopassi, 2007; Napoli et al., 2006; Pandolfo, 1999; Parent et al., 2015; Perdomini et al., 2014; Poburski et al., 2016; Rötig et al., 1997; Söderberg et al., 2016; Stemmler et al., 2010). FeS clusters are important for the function of mitochondrial respiratory chain complexes I, II and III, as well as several other enzymes (Lill et al., 2014; Lim et al., 2013; Söderberg et al., 2016). Frataxin-depleted cells show abnormal FeS cluster formation, decreased activities of FeS cluster-containing proteins, iron accumulation in the mitochondrial matrix, increased reactive oxygen species (ROS) production, and impairment of the electron transport chain, leading to reduced ATP production (Lu and Cortopassi, 2007; Marmolino et al., 2010; Poburski et al., 2016) in FRDA patients and animal models.

Expression of PGC-1α (PPARGC1A), the mitochondrial biogenesis master regulator, has been studied in FRDA patient samples. Paradoxical findings of PGC-1α upregulation and downregulation were found in different FRDA patient fibroblast lines (García-Giménez et al., 2011; Marmolino et al., 2010). Functional genome analysis shows downregulation of PGC-1α mRNA in FRDA patient lymphoblastic cell lines, primary skin fibroblasts and skeletal muscle, but a trend of upregulation in the heart (Coppola et al., 2009). These findings might reflect differential regulation at different disease stages and in different tissues. Biopsies of affected tissues (cardiac tissue, nervous system) from patients and postmortem findings generally represent only the alterations at advanced and end stages of the disease (Koeppen et al., 2011, 2015a,b, 2016; Kruger et al., 2016). Patient lymphocytes, platelets and fibroblasts are not affected clinically (Coppola et al., 2011; García-Giménez et al., 2011; Morán et al., 2010; Salehi et al., 2014). Thus, the relevance of peripheral findings to neurological events is unclear.

Examination of the pathophysiological changes in mouse models at asymptomatic ages can be used to predict the human phenotype. Complete frataxin knockout in mouse models is prenatally lethal, whereas GAA repeat expansions in FRDA patients result in decreased frataxin levels, to 2-20% of those of healthy controls (Lazaropoulos et al., 2015). Neuron-specific knockouts have an early onset phenotype that resembles fully developed changes of FRDA (Cossee et al., 2000; Simon et al., 2004); however, these models are too severe to appreciate the earliest features of the disorder. Identification of early changes thus requires a model in which the phenotype is present, but slowly evolving in the same manner as FRDA progresses. The frataxin knock-in/knockout (KIKO) mouse model of FRDA exhibits these features. It has a knock-in expanded GAA repeat on one allele (230 GAAs) and knockout of *FXN* on the other allele, leading to mice with moderate overall deficiency of frataxin early in life (20-30% of control levels), comparable to the levels in mildly affected patients. No overt neuronal loss appears in initial studies, but mRNA panels from tissue share many features with those from patients (Miranda et al., 2002). More sophisticated studies in KIKO mice identify significant neurobehavioral deficits in inverted screen, treadscan and Von Frey tasks at >8 months of age, resembling clinical manifestations of cerebellar gait ataxia, decreased peripheral sensitivity, and decreased motor strength and endurance in late-onset FRDA patients (McMackin et al., 2016). Thus, the KIKO mouse constitutes a suitable model to search for early pathophysiological changes of FRDA by examining its physiological and biochemical properties at asymptomatic ages (1, 3 and 6 months of age). In the present study, we identify early impaired PGC-1α-associated mitochondrial biogenesis pathways as a potential mediator of cerebellum dysfunction and ataxia, thereby providing potential pathogenic mechanisms and therapeutic targets for early intervention in FRDA patients.

## RESULTS

### Early impaired mitochondrial biogenesis pathways in the cerebellum of frataxin KIKO mice

**Fig. 1.**
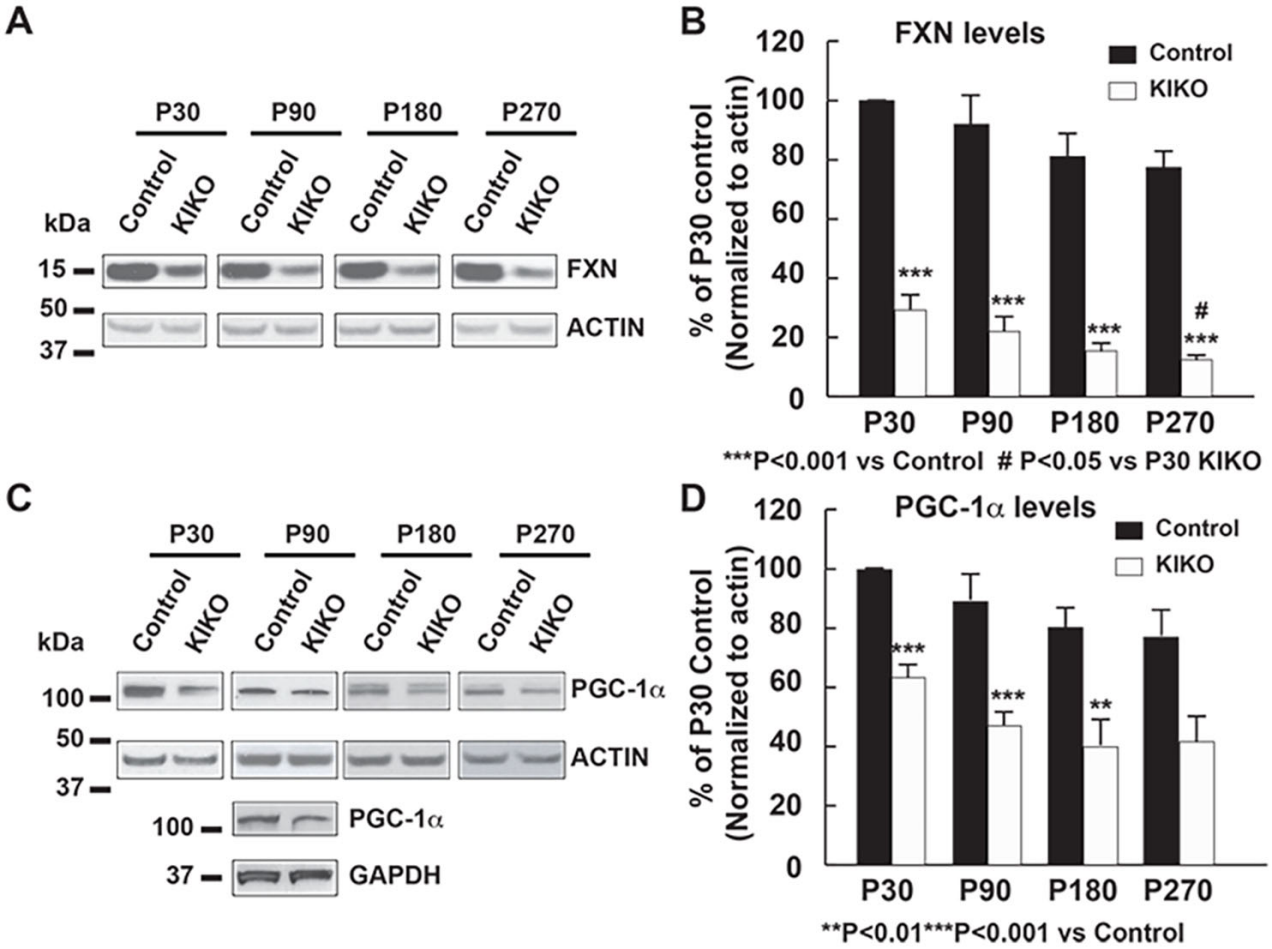
**Levels of the mitochondrial biogenesis master regulator PGC-1α are significantly decreased in the cerebellum of frataxin-deficient KIKO mice at asymptomatic and symptomatic ages.** Western blotting of cerebellar homogenates (30 μg per lane) showing frataxin (A,B) and PGC-1α (C,D) levels, as well as actin as an internal control, in the cerebellum of KIKO mice and age-matched controls at postnatal days P30, P90, P180 and P270 (*n*=3-8 for KIKO and control mice per time point; ^#^*P*<0.05, **P<0.01, ***P<0.001; two-tailed, unpaired Student's *t*-test). Blots in Fig. 1 were stripped and reprobed with multiple antibodies in Figs 2 and 6; α-actin served as the loading control for each.

We first examined the levels of frataxin and the mitochondrial biogenesis master regulator PGC-1α in cerebellar homogenates of KIKO mice at both asymptomatic [postnatal day (P) 30, P90, P180] and symptomatic (P270) ages (McMackin et al., 2016). In wild-type control mice, frataxin and PGC-1α levels are slightly decreased or remain unaltered in cerebellar homogenates at P180 and P270 compared with P30 and P90 (Fig. S1). At all ages, frataxin levels are significantly reduced in cerebellar homogenates of KIKO mice compared with those of age-matched controls (16-29% residual frataxin, *P*<0.001). Moreover, the frataxin levels in KIKO cerebellum progressively decrease over time, with P270 mice having significant lower levels than P30 mice (*P*<0.05) (Fig. 1A,B). Interestingly, the levels of PGC-1α are significantly decreased in cerebellar homogenates of KIKO mice at asymptomatic ages (P30, 37% reduction, *P*<0.001; P90, 47% reduction, *P*<0.001; P180, 50% reduction, *P*<0.01) and remain lower at symptomatic ages (P270, 46% reduction, *P*=0.056) compared with age-matched controls (Fig. 1C,D). This suggests an early impairment of PGC-1α mitochondrial biogenesis pathways in KIKO cerebellum. Downregulation of PGC-1α might thus lead to progressive loss of frataxin in P270 KIKO cerebellum.

**Fig. 2.**
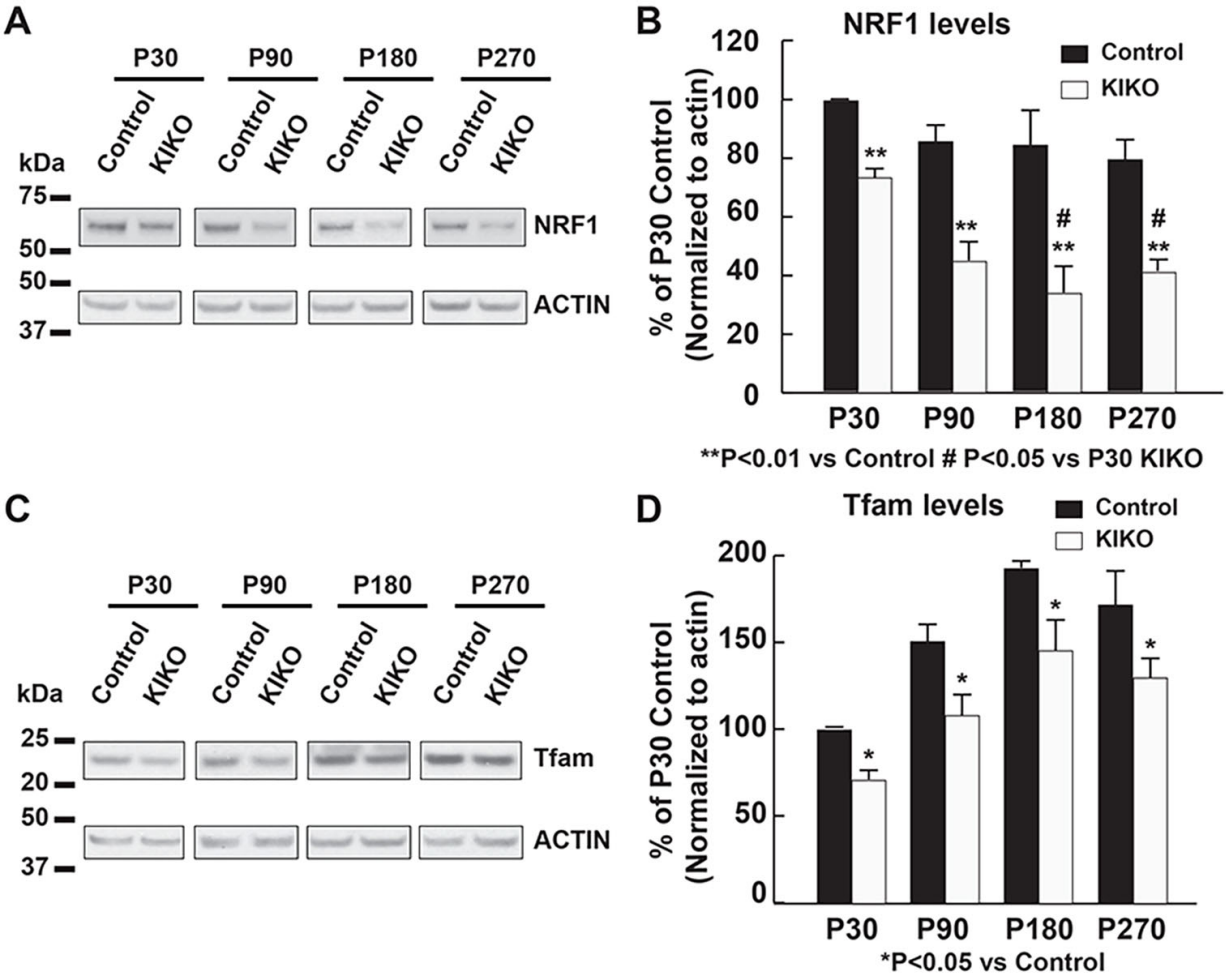
**Levels of PGC-1α effectors NRF1 and Tfam are significantly decreased in KIKO cerebellum at asymptomatic and symptomatic ages.** Western blotting and quantification of cerebellar homogenates (30 μg per lane) showing NRF1 (A,B) and Tfam (C,D) levels, as well as actin as an internal control, in the cerebellum of KIKO mice and controls at P30, P90, P180 and P270 (*n*=3-8 for KIKO and control mice per time point; ^#^*P*<0.05, *P<0.05, **P<0.01; two-tailed, unpaired Student's *t*-test). Blots in Fig. 2 were stripped and reprobed with multiple antibodies in Figs 1, 3 and 6; α-actin served as the loading control for each.

PGC-1α can activate nuclear respiratory factor 1 (NRF1), leading to the transcription of both nuclear-encoded mitochondrial proteins and the mitochondrial transcriptional factor Tfam. Tfam then activates transcription and replication of the mitochondrial genome, thereby controlling mitochondrial biogenesis (Gleyzer et al., 2005; Scarpulla et al., 2012; Ventura-Clapier et al., 2008). We thus examined the levels of NRF1 and Tfam in cerebellar homogenates of KIKO mice compared with controls. In wild-type control mice, NRF1 levels in cerebellar homogenates are slightly decreased or remain unaltered at P180 and P270 compared with P30 and P90 (Fig. S2A), whereas Tfam levels are increased at P90, P180 and P270 compared with P30 (Fig. S2B), which is consistent with previous findings on age-related increases in Tfam and mitochondrial DNA (mtDNA) in rat cerebellum (Dinardo et al., 2003). Similar to PGC-1α, the levels of NRF1 are significantly decreased in cerebellar homogenates of KIKO mice at both asymptomatic and symptomatic ages compared with controls (22%, 50%, 52% and 45% reduction at P30, P90, P180 and P270, respectively, *P*<0.01). Noticeably, the NRF1 levels in KIKO cerebellum at P180 and P270 are significantly lower than those at P30 (*P*<0.05) (Fig. 2A,B), suggesting progressive downregulation of the PGC-1α/NRF1 pathways in KIKO cerebellum. Furthermore, levels of the mitochondrial transcriptional factor Tfam are moderately, but significantly, decreased in KIKO cerebellar homogenates at both asymptomatic (29%, 28% and 24% reduction at P30, P90 and P180, respectively, *P*<0.05) and symptomatic (23% reduction at P270, *P*<0.05) ages compared with age-matched controls (Fig. 2C,D). Our findings thus demonstrate early impairment of PGC-1α/NRF1/Tfam mitochondrial biogenesis pathways in KIKO cerebellum, and suggest that impairment of mitochondrial biogenesis is an early event leading to cerebellar dysfunction in KIKO mice.

### Early cerebellar mitochondrial deficiency in asymptomatic KIKO mice

**Fig. 3.**
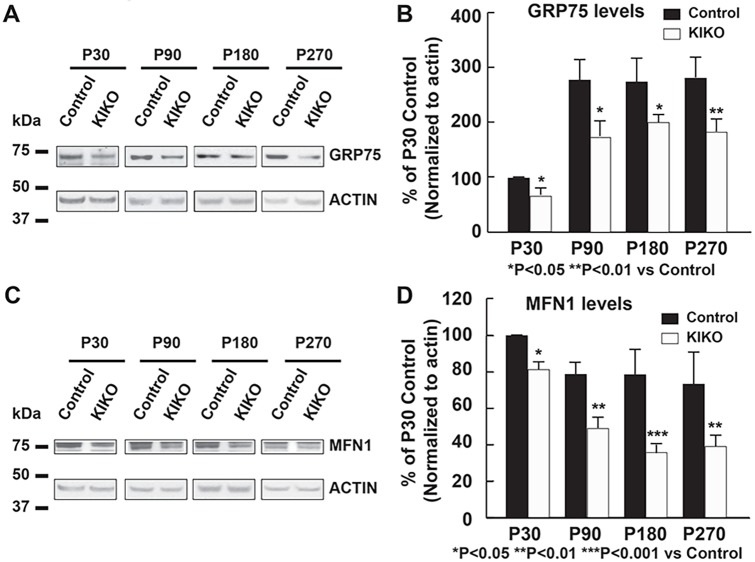
**Levels of the mitochondrial markers GRP75 and MFN1 are significantly decreased in KIKO cerebellum at symptomatic and symptomatic ages.** Western blotting and quantification of cerebellar homogenates (30 μg per lane) showing GRP75 (A,B) and MFN1 (C,D) levels, as well as actin as an internal control, in the cerebellum of KIKO mice and controls at P30, P90, P180 and P270 (*n*=3-8 for KIKO and control mice per time point; *P<0.05, **P<0.01 ***P<0.001; two-tailed, unpaired Student's *t*-test). Blots in Fig. 3 were stripped and reprobed with multiple antibodies in Figs 2 and Fig 6; α-actin served as the loading control for each.

To determine whether mitochondrial deficiency occurs in KIKO mouse cerebellum, we examined the levels of the mitochondrial markers GRP75 (HSPA9) and mitofusin-1 (MFN1) in cerebellar homogenates of P30, P90, P180 and P270 mice. In wild-type control mice, GRP75 levels in cerebellar homogenates are markedly increased at P90, P180 and P270 compared with P30 (Fig. S3A), whereas MFN1 levels are slightly decreased at P180 and P270 compared with P30 and P90 (Fig. S3B). GRP75 levels are significantly decreased in KIKO mice at asymptomatic (34%, 37% and 27% reduction at P30, P90 and P180, respectively, *P*<0.05) and symptomatic (35% reduction at P270, *P*<0.01) ages compared with age-matched controls (Fig. 3A,B). In addition, MFN1 levels are also decreased in cerebellar homogenates of KIKO mice at asymptomatic (21%, 48% and 46% reduction at P30, P90 and P180, respectively, *P*<0.05) and symptomatic (33% reduction at P270, *P*<0.01) ages compared with age-matched controls (Fig. 3C,D).

**Fig. 4.**
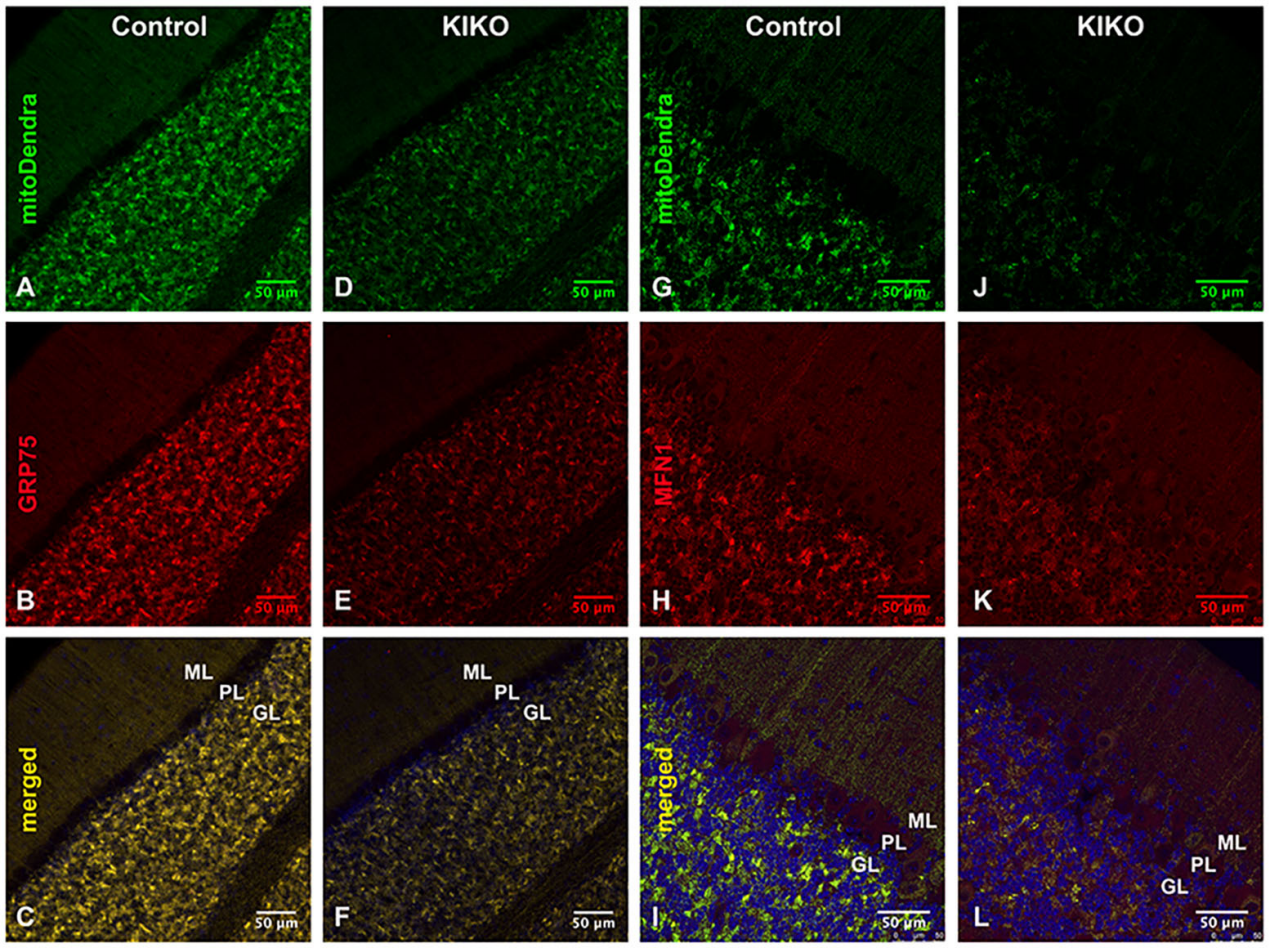
**Reduction of GRP75 and MFN1 in the cerebellar cortex of asymptomatic mitoDendra-KIKO mice.** (A-F) Confocal images of mitoDendra (green), GRP75 (red) and merged images with DAPI-stained nuclei, showing a reduction in the overall levels of mitoDendra and GRP75 in the cerebellar cortex of mitoDendra-KIKO mice (D-F) compared with control mice (A-C) at P90. (G-L) Confocal images of mitoDendra (green), MFN1 (red) and merged images with DAPI-stained nuclei, showing a reduction in the overall levels of MFN1 and mitoDendra in the cerebellar cortex of mitoDendra-KIKO mice (J-L) compared with control mice (G-I) at P90. GL, granular layer; ML, molecular layer; PL, Purkinje layer. Scale bars: 50 μm.

**Fig. 5.**
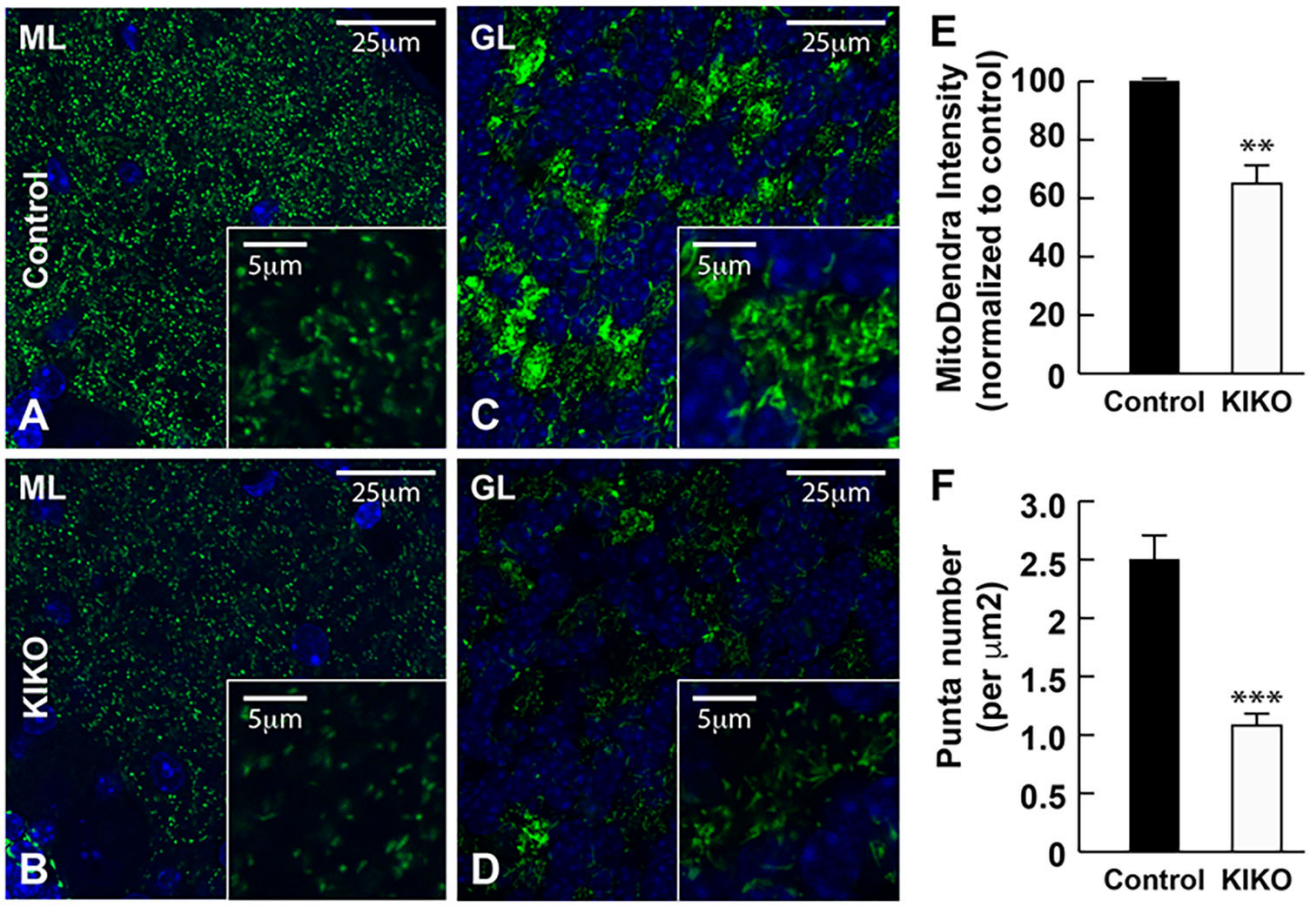
**Levels and number of mitoDendra are significantly reduced in the cerebellar cortex of asymptomatic mitoDendra-KIKO mice.** (A-D) Confocal images of mitoDendra (green) merged images with DAPI-stained nuclei, showing marked reduction in the number of Dendra-labeled mitochondria in the cerebellar ML (B) and GL (D) of P90 KIKO mice compared with control mice (A,C). Insets are higher magnification confocal images, showing marked reduction of Dendra-labeled mitochondrial puncta in the cerebellar ML (B) and GL (D) of mitoDendra-KIKO mice compared with control mice (A,C). (E,F) Quantification of levels and number of mitoDendra puncta per μm^2^, showing a significant decrease in the levels and number of mitoDendra in the cerebellar cortex of mitoDendra-KIKO mice compared with control mice (E) (*n*=5 sections per animal; three animals per group; ***P<0.001, two-tailed, unpaired Student's *t*-test). Scale bars: 25 μm (5 μm in insets).

To investigate mitochondrial deficiency in the intact KIKO cerebellum, we crossbred the KIKO mouse with a transgenic mouse expressing fluorescent Dendra-labeled mitochondria in the nervous system (mitoDendra mouse) (Magrané et al., 2014). The mitoDendra transgene allows the study of mitochondrial changes in KIKO mice, and provides a marker of mitochondrial location relative to detailed cerebellar anatomy. Dendra-labeled mitochondria are widely distributed and colocalize with frataxin in the cerebellar cortex. In KIKO mice carrying a mitoDendra transgene (mitoDendra-KIKO mice), the overall levels of frataxin immunoreactivity are markedly reduced in the cerebellar cortex compared with controls at P90 (H.L., J.M., E.M.C. and D.R.L., unpublished). The overall levels of mitoDendra are reduced in KIKO mice (Fig. 4D,J versus A,G), and the GRP75 and MFN1 immunoreactivities colocalize with mitoDendra and are dramatically reduced in cerebellar cortex of mitoDendra-KIKO mice (Fig. 4D-F,J-L), compared with age-matched controls (Fig. 4A-C,G-I), suggesting cerebellar mitochondrial deficiency in asymptomatic mitoDendra-KIKO mice. Higher magnification confocal images show the overall reduction of Dendra-labeled mitochondria in cerebellar molecular (ML) and granular (GL) layers of mitoDendra-KIKO mice (Fig. 5B,D, insets), compared with age-matched controls (Fig. 5A,C, insets), at P90 (Fig. 5). Quantification shows that both fluorescence levels and number of mitoDendra puncta are significantly reduced in the cerebellar cortex of KIKO mice compared with controls (Fig. 5E,F; *P*<0.01, *P*<0.001, respectively), further confirming early impairment of mitochondrial biogenesis in asymptomatic KIKO mouse cerebellum.

### Mitochondrial complex I, II and IV deficiency in the cerebellum of asymptomatic KIKO mice

**Fig. 6.**
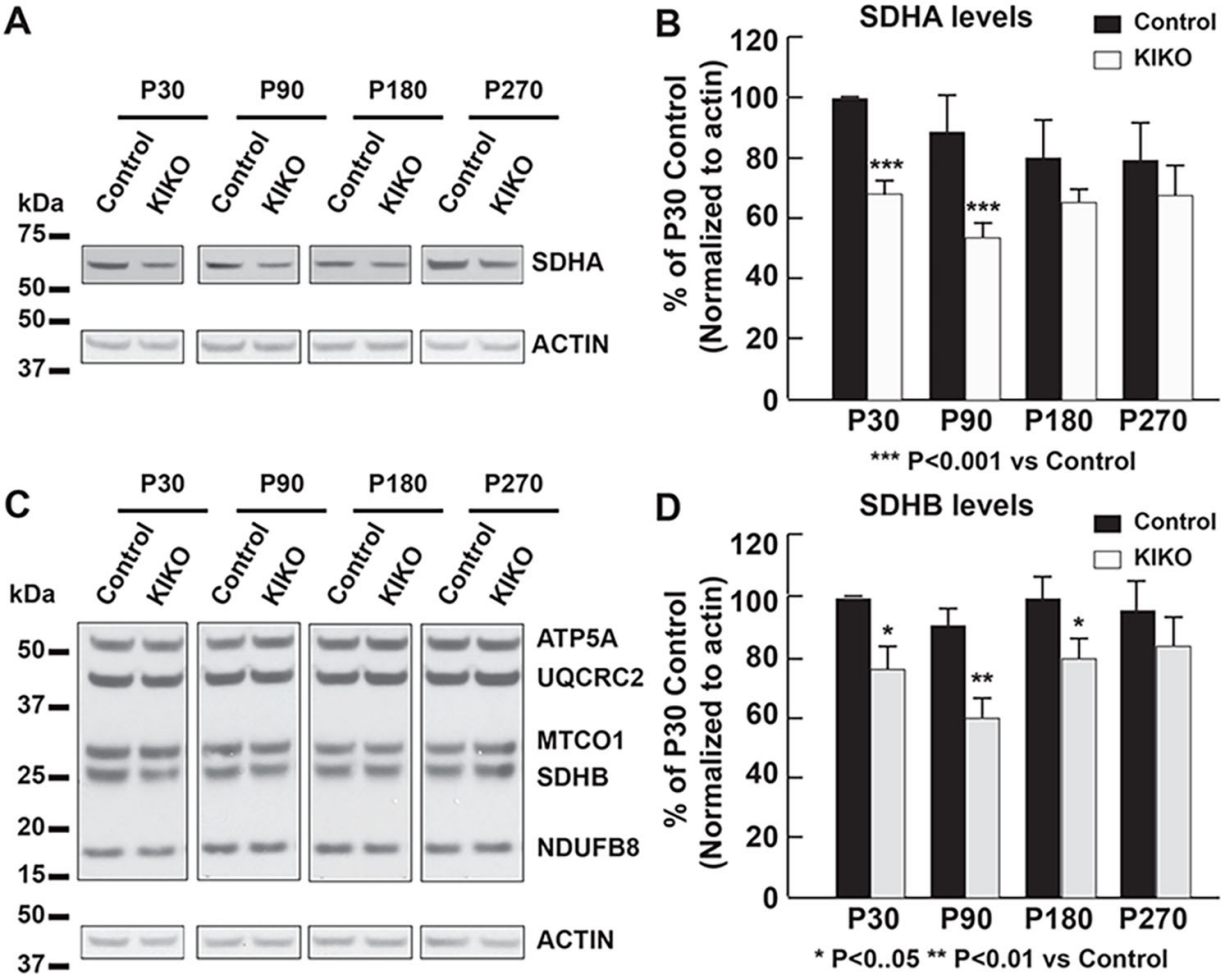
**Mitochondrial complex I and II subunits levels are significantly reduced in the cerebellum of asymptomatic KIKO mice.** Western blotting and quantification of cerebellar homogenates (30 μg per lane), showing complex II subunit SDHA (A,B) and complex I-V markers NDUFB8, SDHB, UQCRC2, MTCO1 and ATP5A (C,D) levels, as well as actin as an internal control, in the cerebellum of KIKO mice and controls at P30, P90, P180 and P270 (*n*=3-8 for KIKO and control mice per time point; *P<0.05, **P<0.01, ***P<0.001; two-tailed, unpaired Student's *t*-test). Blots in Fig. 6 were stripped and reprobed with multiple antibodies in Figs 1-3; α-actin served as the loading control for each.

Deficiencies in the oxidative phosphorylation (OXPHOS) system, including abnormalities in complex I and II, have been found in FRDA patients (Rötig et al., 1997; Salehi et al., 2014). Thus, we first examined the relative abundance of several respiratory chain subunits in KIKO mouse cerebellar homogenates (Fig. 6). Levels of complex II subunits SDHA (Fig. 6A,B) and SDHB (Fig. 6C,D) are significantly reduced in KIKO mice at P30 and P90 (32% and 39% reduction, respectively, for SDHA, *P*<0.001; 24% and 33% reduction for SDHB, *P*<0.05 and *P*<0.01, respectively). Although levels of the complex I core subunit NDUFB8 are moderately, but significantly, decreased in early asymptomatic KIKO cerebellum (P30, 12%, *P*<0.05; P90, 22% reduction, *P*<0.05), the levels of UQCRC2 (complex III), MTCO1 (complex IV) and ATP5A (ATP5A1, complex V) are only slightly decreased or remain unaltered (Fig. 6C,D). Noticeably, deficiency of complex II subunit levels at early asymptomatic ages (P30, P90) appears to be compensated at P180 and P270 (Fig. 6C,D), suggesting a compensatory response of some components of the OXPHOS system in KIKO mouse cerebellum.

**Fig. 7.**
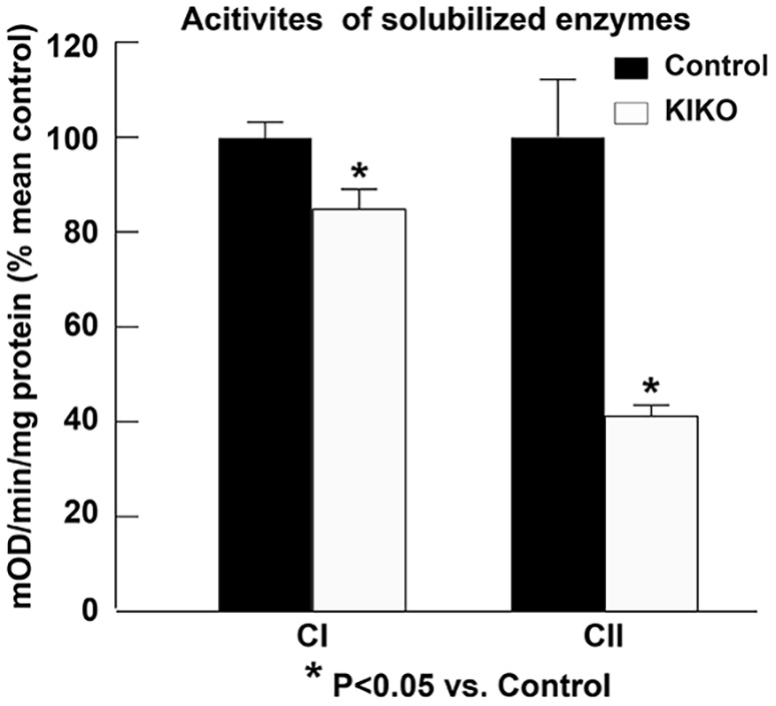
**Mitochondrial complex I and II activities are compromised in the cerebellar homogenates of asymptomatic KIKO mice.** Complex I and II activity assay in the cerebellar homogenates of KIKO mice compared with control mice at P90. All activity values are expressed as percentages of mean control values (CI, *n*=4 mice per group; CII, *n*=3 mice per group; *P<0.05, two-tailed, unpaired Student's *t*-test).

**Fig. 8.**
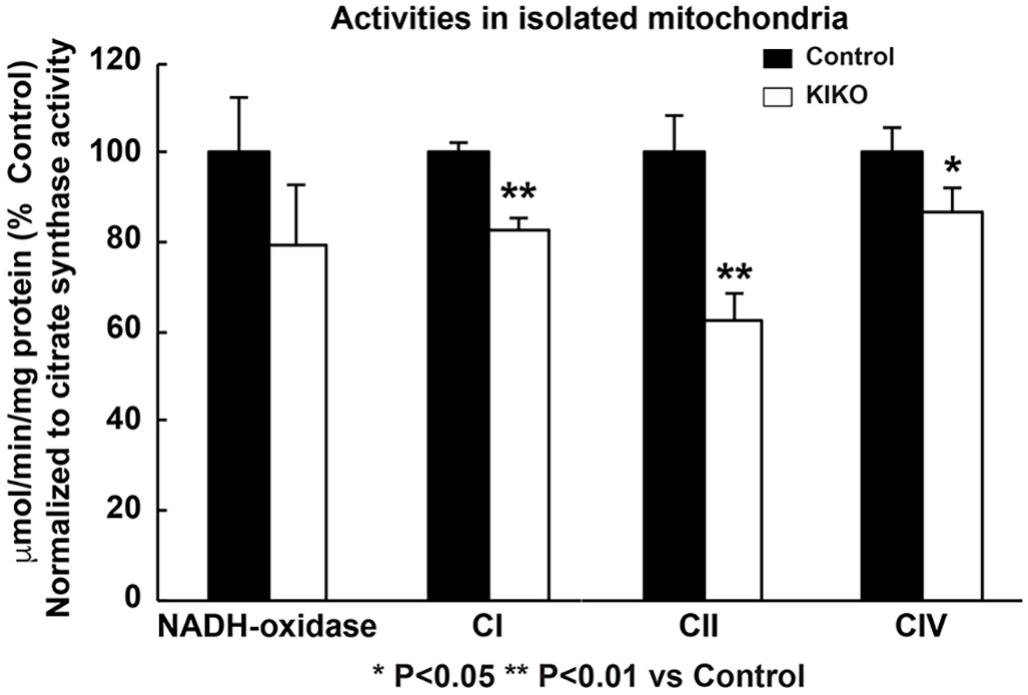
**Reduction of complex I, II and IV activities in isolated mitochondria from the cerebellum of asymptomatic KIKO mice.** Mitochondria were isolated from the cerebellum of P90 mice. Activities of several mitochondrial respiratory chain complexes in the cerebellum of KIKO and control mice were measured. All activity values were normalized to citrate synthase and expressed as percentages of those of control mice (*n*=3 mice per group; *P<0.05, **P<0.01, two-tailed, unpaired Student's *t*-test).

We then assessed respiratory chain complexes' activities using two preparations: solubilized enzymes (Fig. 7) and isolated mitochondria (Fig. 8). Complex I activity is moderately, but significantly, reduced in P90 KIKO cerebellar homogenates when compared to controls (Fig. 7, 15% reduction, *P*<0.05), while complex II activity is dramatically and significantly decreased (Fig. 7, 59% reduction, *P*<0.05). In isolated mitochondria, measurement of NADH oxidase (NADH oxidase activity is a result of full activity of CI+CIII+CIV) was not significantly reduced. However, NADH: hexammineruthenium (HAR) oxidoreductase activity was significantly reduced in KIKO cerebellum (*P*<0.01), by 15% (Fig. 8), similar to the results in Fig. 7. Measurement of succinate dehydrogenase (CII activity) revealed a 38% reduction in complex II activity in KIKO cerebellum, compared to controls (*P*<0.01) (Fig. 8), similar to the results in Fig. 7. Finally, measurement of ferrocytochrome *c* oxidase (CIV activity) demonstrated a significant decrease in complex IV activity in KIKO cerebellum (*P*<0.05) (Fig. 8). Unlike the other measured activities, reduction of complex IV activity was preserved in P270 KIKO mice (data not shown; *n*=2 mice in KIKO and control groups; *P*<0.01). Our findings thus demonstrate early mitochondrial respiratory chain complex deficiencies in asymptomatic KIKO mouse cerebellum, consistent with changes seen in FRDA patients (Rötig et al., 1997; Salehi et al., 2014).

## DISCUSSION

The present study uses a frataxin-deficient FRDA mouse model (KIKO mouse) to demonstrate early impairment of mitochondrial biogenesis and respiratory chain complex I, II and IV deficiencies in cerebellar cortex at asymptomatic ages. Examination of mitochondrial biogenesis pathways shows significant reduction in levels of the mitochondrial biogenesis master regulator PGC-1α and its downstream effectors NRF1 and Tfam, as well as mitochondrial markers GRP75 and MFN1, suggesting early impairment of cerebellar mitochondrial biogenesis. There was also a significant reduction in Dendra-labeled mitochondria in asymptomatic KIKO mice carrying a mitoDendra transgene, further confirming early mitochondrial deficiency. Moreover, compromised mitochondrial complex enzyme activities and levels match those in FRDA patients (Rötig et al., 1997; Salehi et al., 2014). Our findings thus identify early impaired mitochondrial biogenesis as a potential mediator leading to cerebellar dysfunction and ataxia in this FRDA model.

PGC-1α controls the expression of the mitochondrial respiratory chain and the biogenesis of mitochondria (Koopman et al., 2010; Scarpulla et al., 2012; Ventura-Clapier et al., 2008; Villena, 2015). The nuclear respiratory factor NRF1 has been identified as a target for PGC-1α induction of mitochondrial biogenesis. PGC-1α activation of NRF1 leads to transcription of nuclear-encoded mitochondrial genes and of the mitochondrial DNA transcriptional factor Tfam, which activates transcription and replication of the mitochondrial genome, thereby controlling mitochondrial biogenesis (Gleyzer et al., 2005; Koopman et al., 2010; Scarpulla et al., 2012; Ventura-Clapier et al., 2008). PGC-1α dysregulation and impaired mitochondrial biogenesis have been implicated in neurodegenerative disorders (Calkins et al., 2011; Coppola et al., 2009; García-Giménez et al., 2011; Jiang et al., 2016; Johri et al., 2013; Marmolino et al., 2010; McGill and Beal, 2006; Reddy et al., 2012; Ruiz et al., 2012; Sandi et al., 2014; Shin et al., 2011; Stevens et al., 2015; Thau et al., 2012). Downregulation of PGC-1α mRNA and protein have been reported in skeletal muscle, fibroblasts and neural precursor cells cultured from FRDA patients and animal models (Coppola et al., 2009; Marmolino et al., 2010; Sandi et al., 2014). Paradoxical upregulation of PGC-1α mRNA and protein was also reported in fibroblasts from two FRDA patients (García-Giménez et al., 2011). Our results demonstrate downregulation of the PGC-1α/NRF1/Tfam pathway in KIKO cerebellum at asymptomatic and symptomatic ages, suggesting that early impaired mitochondrial biogenesis associated with PGC-1α deficiency is an upstream event leading to cerebellar dysfunction in FRDA patients. Interestingly, a recently published paper shows downregulation of mitochondrial biogenesis markers at transcriptional levels, including NRF1 mRNA, Tfam mRNA and mitochondrial DNA, in FRDA patient fibroblasts and blood, as well as in KIKO mouse brain (Jasoliya et al., 2017). This is consistent with our findings in KIKO mouse cerebellum, an affected tissue in FRDA. Furthermore, reduction of the mitochondrial protein GRP75 is found in KIKO cerebellum. GRP75 was previously demonstrated to be a binding partner of frataxin stabilizing the FeS complex (Shan and Cortopassi, 2012, 2016). Thus, one possible hypothesis is that frataxin deficiency leads to the deficiency of GRP75 and FeS complexes, resulting in mitochondrial biogenesis deficits. An alternative hypothesis is that frataxin deficiency leads to PGC-1α-mediated mitochondrial biogenesis deficits, causing the decrease in the mitochondrial protein GRP75. Jasoliya et al. (2017) demonstrated that frataxin deficiency causes a mitochondrial biogenesis defect in multiple paradigms, including FDRA patient fibroblasts and blood, normal fibroblasts after frataxin knockdown, and KIKO mouse brain, and frataxin expression rescues these features of mitochondrial biogenesis deficiency. Our findings support the hypothesis that frataxin deficiency leads to PGC-1α-mediated mitochondrial biogenesis deficits, resulting in the deficiencies of mitochondria and mitochondrial proteins. Using KIKO mice carrying the mitoDendra transgene, we show significant reduction in the fluorescent level and number of Dendra-labeled mitochondria in the cerebellum of asymptomatic KIKO mice, suggesting that early impairment of PGC-1α-mediated mitochondrial biogenesis as a potential mediator of cerebellar dysfunction and ataxia in FRDA patients. Indeed, conditional deletion of PGC-1α in parvalbumin-positive neurons leads to degeneration of cerebellar Purkinje neurons and the ataxic phenotype in mice (Lucas et al., 2014), supporting an important pathophysiological role for PGC-1α-mediated mitochondrial biogenesis in cerebellar normal function and dysfunction.

Mitochondrial biogenesis deficits have been found in many neurodegenerative diseases, including Alzheimer's disease, Parkinson's disease, amyotrophic lateral sclerosis, multiple sclerosis, Huntington's disease and stroke, as well as inherited mitochondrial DNA mutation diseases, such as Leber's hereditary optic neuropathy (LHON), and the syndrome of neurogenic muscle weakness, ataxia and retinitis pigmentosa (NARP/MILS) (Calkins et al., 2011; Giordano et al., 2014; Golpich et al., 2017; Hayashi et al., 2017; Onyango et al., 2010; Uittenbogaard and Chiaramello, 2014; Wojewoda et al., 2011). This is implicated as a common pathogenic mechanism in neurodegeneration. Our findings suggest early mitochondrial biogenesis deficits as a potential mediator of cerebellar dysfunction in FRDA, and thus as a potential therapeutic target in FRDA and other neurodegenerative diseases. Administration of PGC-1α activators reduces pathology in multiple neurodegenerative diseases (Aleshin et al., 2013; Katsouri et al., 2012, 2016, 2011). Additionally, dimethyl fumarate, which is an NRF2 (NFE2L2) activator effective in multiple sclerosis, increases mitochondrial biogenesis in cells, mice and patients with multiple sclerosis, further suggesting that mitochondrial biogenesis deficiency represents a valid target in neurodegenerative disease (Hayashi et al., 2017). In addition to our findings as a potential pathogenic mechanism, mitochondrial biogenesis deficits could also serve as a potential therapeutic target for treatment of neurodegeneration in FRDA.

Neurons are highly energy-demanding cells that depend on the mitochondrial OXPHOS system, which is composed of respiratory complexes I to IV, forming proton-motive force across the inner mitochondrial membrane. Consequently, complex V or ATP synthase uses it to generate ATP for cellular needs (Breuer et al., 2013). Like PGC-1α, mitochondrial OXPHOS dysfunction occurs in a variety of neurodegenerative diseases, including FRDA (Coppola et al., 2009; Johri et al., 2013; Lopez-Gallardo et al., 2011; Mastroeni et al., 2016; McGill and Beal, 2006; Pesini et al., 2014; Salehi et al., 2014; Seelert et al., 2009; Shin et al., 2011; Sweeney and Song, 2016; Thau et al., 2012). A decrease in complex I, II and III activity has been observed in endomyocardial biopsies of two FRDA patients (Rötig et al., 1997), suggesting mitochondrial complex I and II deficiency in FRDA. Gene expression profiling in peripheral blood mononuclear cells (PBMCs) of a small cohort of FRDA patients suggests complex I deficiency, specifically in the mtDNA-encoded ND2, ND4L and ND6 and the nuclear-encoded gene NDUFA1 (Salehi et al., 2014). Frataxin is crucial for FeS cluster formation, which is required for the functional activity of complex I, II, III and aconitase (Cavadini et al., 2002; Martelli et al., 2007; Rötig et al., 1997). Yeast and human frataxin physically interact with complex II subunits SDHA and SDHB, and complex II activity is severely impaired in yeast and *Caenorhabditis elegans* mutants lacking frataxin (Gonzalez-Cabo et al., 2005; Vazquez-Manrique et al., 2006). Our findings show significant reduction of complex I and II levels and complex I, II and IV activities in both tissue homogenates and isolated mitochondria from the cerebellum of asymptomatic P90 KIKO mice. These results indicate that frataxin deficiency might lead to deficiency of complex I, II and IV in FRDA cerebellum. Interestingly, another mouse model of FRDA (Y8GR) has decreased mitochondrial complex I, but overactivation of complex II in the cerebellum. This reported complex activity imbalance leads to ROS generation, resulting in glutathione depletion and increased lipid peroxidation, which contribute to neuronal death in cerebellar granular neurons cultured from Y8GR mice (Abeti et al., 2016). Our and others’ findings thus implicate mitochondrial OXPHOS dysfunction in the pathogenesis of cerebellar dysfunction in FRDA.

PGC-1α activation of NRF1 leads to both transcription of nuclear-encoded mitochondrial OXPHOS subunits and the mitochondrial transcriptional factor Tfam, which activates the transcription of 13 mRNAs for OXPHOS subunits. Both nuclear- and mitochondria-encoded subunits of the respiration chain are assembled in mitochondria (Gleyzer et al., 2005; Koopman et al., 2010; Scarpulla et al., 2012; Ventura-Clapier et al., 2008). Our findings thus suggest that early impairment of PGC-1α/NRF1/Tfam mitochondrial biogenesis pathways could contribute to early respiratory chain complex deficiency in KIKO cerebellum at asymptomatic ages. Reduction of mitochondria-encoded subunit ND1 and ND6 was recently found in KIKO mouse brain and FRDA patient blood (Jasoliya et al., 2017). Mitochondrial biogenesis deficits could thus also serve as a potential biomarker in FRDA patients. Taken together, our findings reveal early mitochondrial biogenesis deficits as a potential pathogenic mechanism, and also as a potential biomarker and therapeutic target in FRDA patients.

## MATERIALS AND METHODS

### Materials

C57BL/6 mice were purchased from Charles River Laboratories and frataxin KIKO mice from Jackson Laboratory (B6.Cg-Fxn^tm1.1Pand^ Fxn^tm1Mkn^/J; stock number 012329). KIKO mice were twice crossbred with Thy1-mitoDendra mice [B6SJL-Tg (Thy1-COX8A/Dendra)57Gmnf/J; stock number 025401] to generate control-mitoDendra and KIKO-mitoDendra mice (Magrané et al., 2014). Antibodies used for western blotting (WB) and immunohistochemistry (IHC) include anti-frataxin (Abcam, ab175402, 1:1000, WB), anti-PGC-1α (Abcam, ab54481, 1:1000, WB), anti-NRF1 (Abcam, ab175932, 1:1000, WB), anti-Tfam (Abcam, ab131607, 1:1000, WB), anti-mitofusin-1 (Novus Biologicals, NBP1-51841, 1:250, IHC and 1:1000, WB), anti-GRP75 (Abcam, ab2799, 1:250, IHC; 1:1000, WB), anti-SDHA (Cell Signaling Technology, #5839, 1:1000, WB), Total OXPHOS Rodent WB Antibody Cocktail (Abcam, ab110413, 1:500, WB), anti-GAPDH (Novus Biologicals, NB300-221, 1:1000, WB) and anti-actin (Abcam, ab3280, 1:5000, WB). All animals were treated according to the protocols approved by The Children's Hospital of Philadelphia Institutional Animal Care and Use Committee and Weill Cornell Medical College Institutional Animal Care and Use Committee.

### Tissue preparation and immunohistochemistry

For tissue homogenate preparation, cerebella of KIKO mice and age-matched heterogeneous controls [both wild-type/wild-type (WTWT) and knock-in/wild-type (KIWT) mice] at postnatal days P30, P90, P180 and P270, ±10 days, of either sex were harvested. KIWT mice are the equivalent to human heterozygous carriers. The tissues were mechanically homogenized in 20 ml lysis buffer per 1 g weight, and lysed for 1 h at 4°C. Lysis buffer contained 150 mM NaCl, 1 mM EDTA, 100 mM Tris-HCl, 1% Triton X-100 and 1% sodium deoxycholate, pH 7.4, supplemented the day of use with 1:500 EDTA-free protease inhibitor cocktail III (Calbiochem, 53914). Debris was cleared by centrifugation at 39,000 ***g*** for 1 h at 4°C. Supernatants were stored at −80°C until use.

For immunohistochemical studies, KIKO-mitoDendra mice and age-matched knockout/wild-type (KOWT)-mitoDendra controls of either sex at P90 were perfused with 4% paraformaldehyde and the cerebella harvested. KOWT mice were used as controls and are equivalent to the hemizygous carriers. A series of brain coronal floating sections (50 µm) were obtained using a vibratome (VT1200S; Leica, Deerfield, IL) in PBS and stored in PBS with 30% glycerol (vol/vol) and 30% ethylene glycerol (vol/vol) at −20°C. The floating sections were blocked with 5% normal goat serum and 1% bovine serum albumin in combination with 0.3% (vol/vol) Triton X-100 in PBS at room temperature for 1 h, then incubated with primary antibodies at 4˚C overnight and then secondary antibodies conjugated to Alexa Fluor 488 (Invitrogen, A11029) or 568 (Invitrogen, A11036) at room temperature for 60-90 min. Following several washes with PBS, the stained sections were mounted on slides with Vectashield with DAPI (Vector Laboratories, H-1200).

### Western blotting

Western blotting was performed as described previously (Lin et al., 2014a,b). Protein content was determined using a BCA Protein Assay Kit (Thermo Fisher Scientific, 23228). Equal amounts of total protein (30 µg tissue homogenate per lane) were subjected to 4-12% NuPAGE gel for electrophoresis and transferred to nitrocellulose membranes. Membranes were blocked with 3% nonfat milk and incubated with primary antibody overnight at 4°C. Blots were washed 3×10 min in TBST, then incubated with appropriate horseradish peroxidase (HRP)-conjugated secondary antibodies (Cell Signaling Technology) for 2 h at room temperature, and then washed 3×10 min. Reaction bands were visualized using a luminol-enhanced chemiluminescence (ECL) HRP substrate (Thermo Fisher Scientific). Each blot was then incubated with stripping buffer [2% SDS, 50 mM Tris-HCl (pH 6.8) and 100 mM β-mercaptoethanol] for 45 min at room temperature, and reprobed for other proteins, including actin or GADPH used as internal controls. Reaction product levels were quantified by scanning densitometry using ImageJ software (https://imagej.nih.gov/ij/) and normalized to the levels of actin or GAPDH (Lin et al., 2014a,b).

### Respiratory chain complex enzyme activity assay

Mitochondrial complex I and II enzyme activities in cerebellar homogenates of KIKO mice were determined using complex I and II enzyme activity microplate assay kits (Abcam, ab109721 and ab109908) according to the manufacturer's instructions. Briefly, tissues were suspended in 500 µl-1 ml ice-cold PBS and homogenized with a Dounce homogenizer sitting on ice with 20-40 passes, or until the sample was fully homogenized and completely smooth. The protein concentration in the homogenates was determined by BCA assay and adjusted to 5.5 mg/ml with PBS. Protein was further extracted by adding 10× detergent solution to the sample to a final dilution of 1/10 and by incubating on ice for 30 min to allow solubilization. Samples were then centrifuged for 20 min at 4°C at 12,000-16,000 ***g***, and the supernatants collected for use in the enzyme activity assays.

For the complex I activity assay, 200 µl sample was loaded on a microplate coated with a monoclonal antibody against complex I, and incubated for 3 h at room temperature. The microplate was then emptied and twice rinsed with buffer. Assay solution [200 µl, including NADH and dye, extinction coefficient for dye (ε)=25.9 mM^−1^/well] was added to each well and measured at 450 nm every minute for 30 min. Complex I activity in each well was proportional to the increase in absorbance at 450 nm within each well. The activity was expressed as the change in absorbance per minute per amount of sample loaded into the well.

For the complex II activity assay, 200 µl sample was loaded on a microplate coated with a monoclonal antibody against Complex II, and incubated for 2 h at room temperature. The microplate was then emptied and rinsed with buffer. Phospholipid mix (40 µl) was added to each well, and incubated for 30 min at room temperature. Activity solution, which included ubiquinone, succinate and DCPIP, was added to each well. Measurements were taken at 600 nm on a spectrophotometer every 20 s for 60 min. The reduction of ubiquinone and subsequent reduction of DCPIP was measured as a decrease in absorbance at 600 nm. The rate of decrease in absorbance at 600 nm was monitored over time and calculated between two time points for all the samples in which the decrease in absorbance was the most linear. Rate (mOD/min) was calculated as Absorbance 1–Absorbance 2/Time (min), and the activity of immunocaptured complex II as the mean of measurements obtained with immunocaptured enzyme minus the rate obtained without immunocaptured enzyme.

### Mitochondria isolation and complexes activity measurements

Mitochondria were isolated by a standard method of differential centrifugation. Particular care was taken to cool down all media, glassware and centrifuge rotor. Half of the cerebellum was homogenized in 0.5-0.6 ml MSE buffer (225 mM mannitol, 75 mM sucrose, 5 mM HEPES, 0.1% BSA, 1 mM EGTA, 0.1 mM EDTA, pH 8.0) using a 2 ml tight Kontes™ Dounce homogenizer, 60 strokes. Tissue debris was discarded after centrifugation for 4 min at 1500 ***g***. The supernatant was centrifuged for 15 min at 20,000 ***g***. and the membrane pellet was rinsed twice with SET medium [50 mM Tris-HCl (pH 7.5), 0.25 M sucrose, and 0.2 mM EDTA] containing 0.1% BSA. The mitochondrial pellet was resuspended in 60 µl of the same buffer, aliquoted, frozen in liquid nitrogen and stored at −80°C until use for activity measurements.

All activities were measured spectrophotometrically using SpectraMax plate reader (Molecular Devices) in KCl buffer (125 mM KCl, 14 mM NaCl, 20 mM HEPES, 0.2 mM EGTA, pH 7.2). NADH-oxidase, NADH: HAR oxidoreductase (CI), ferrocytochrome *c* oxidase (CIV), and succinate: DCIP reductase (CII) activities were measured, as described (Stepanova et al., 2016). Briefly, NADH-dependent enzymatic activities of complex I were assayed as a decrease in absorption at 340 nm (ε_340nm_=6.22 mM^−1^ cm^−1^) with 150 μM NADH in KCl buffer supplemented with 15 µM cytochrome *c* for NADH-oxidase or with 1 mM KCN and 1 mM HAR for NADH: HAR activity. It was necessary to permeabilize mitochondria with 1 mM MgCl_2_ and 30 µg/ml alamethicin for measuring NADH-dependent activities. The NADH oxidase reaction was >90% sensitive to rotenone. Complex IV activity was measured spectrophotometrically as oxidation of 50 µM ferrocytochrome *c* at 550 nm (ε_550nm_=21.0 mM^−1^ cm^−1^) in KCl buffer supplemented with 0.025% dodecylmaltoside. Ferrocytochrome *c* oxidase activity was fully sensitive to cyanide.

Citrate synthase (CS) activity was measured as an increase in absorption at 412 nm (ε_412nm_=14.2 mM^−1^ cm^−1^) in 20 mM HEPES buffer (pH 7.8) containing 0.1 mM DTNB, 0.4 mM Acetyl-CoA, 0.4 mM oxaloacetate and ∼0.05-0.1 mg/ml mitochondrial protein. Succinate: DCIP reductase activity of complex II was assayed as a decrease in absorption at 600 nm (ε_600nm_=21 mM^−1^ cm^−1^) in 20 mM HEPES buffer (pH 7.8) containing 10 mM succinate, 50 µM ubiquinone-1 and 80 µM DCIP. The reaction was fully sensitive to the specific CII inhibitor malonate. All activities were measured at 25°C and expressed in µmol substrate/min/mg, normalized by CS activity, and expressed as % of values of control mice. Activity values in control mice were as follows (in µmol substrate/min/mg protein): NADH oxidase 0.61±0.08, HAR 1.00±0.05, CIV 3.66±0.19, CII 0.12±0.01, and CS 0.27±0.01. All assays were measured as triplicates. Protein concentration was assayed using Pierce BCA assay.

### Fluorescence imaging and quantification

Fluorescence images were obtained with an Olympus FluoView and a Leica SP8 laser scanning confocal microscope. Confocal scans were performed of mouse cerebellum, using identical imaging parameters for KIKO mice and controls (KOWT). Control sections were included in all experiments to normalize for expected variations in antibody staining intensity performed on different days. Confocal images were acquired at the focal plane with maximal number of Dendra-labeled puncta or large principle neurons from at least three sections per animal and from at least three animals per group. ImageJ was used to quantify the number of Dendra-labeled puncta in cerebellar cortex or large principle neurons in the DN in the acquired confocal images. Thresholds were set at three standard deviations above the mean staining intensity of six nearby regions in the same visual field. Thresholded images present a fixed intensity for all pixels above the threshold after having removed all of those below, and Dendra-labeled puncta in the thresholded images were quantified (Lin et al., 2014b; Lozada et al., 2012).

### Statistical analysis

Data are shown as the mean±s.e.m. Student's *t*-test was performed to compare two conditions, with significance set at *P*<0.05.

## Supplementary Material

Supplementary information
