# Correction: Early cerebellar deficits in mitochondrial biogenesis and respiratory chain complexes in the KIKO mouse model of Friedreich ataxia (doi: 10.1242/dmm.030502)

**DOI:** 10.1242/dmm.033415

**Published:** 2018-01-01

**Authors:** Hong Lin, Jordi Magrane, Amy Rattelle, Anna Stepanova, Alexander Galkin, Elisia M. Clark, Yi Na Dong, Sarah M. Halawani, David R. Lynch

An error was published in *Dis. Model. Mech.* (2017). **10**, 1343-1352 (doi: 10.1242/dmm.030502).

The corrected sentence (Introduction section) is below and the original article has been changed correspondingly.

‘Biopsies of affected tissues (cardiac tissue, nervous system) from patients are uncommon and postmortem findings might represent the alterations at later stages of the disease (Koeppen et al., 2011, 2015a,b, 2016; Kruger et al., 2016).’

