# Supplementary material for: Early cerebellar deficits in mitochondrial biogenesis and respiratory chain complexes in the KIKO mouse model of Friedreich ataxia
Source: Dis Model Mech. 2017 Nov 1;10(11):1343–52. doi: 10.1242/dmm.030502 (PMC5719255; doi:10.1242/dmm.030502)
Supplement: Supplementary information [file dmm-10-030502-s1.pdf]

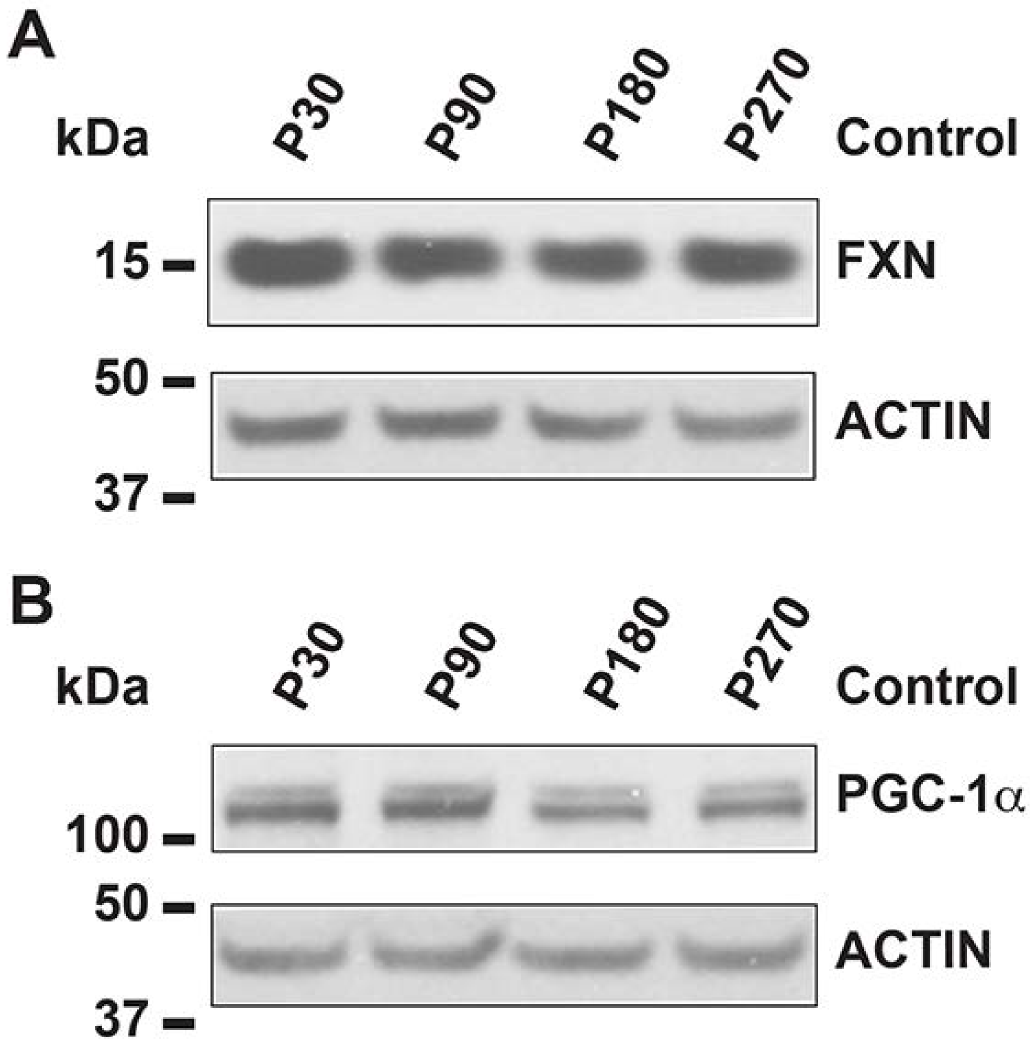

Supplemental Figure 1. Frataxin and PGC-1α expression in cerebellum of P30-P270 wild-type control mice. Western blotting of cerebellar homogenates (30 μg per lane) showing frataxin (**A**) and PGC-1α (**B**) levels as well as actin as an internal control in the cerebellum of wild-type controls at postnatal days P30, P90, P180, and P270 (n=5-8 mice per time-point) .

Disease Models & Mechanisms • Supplementary information

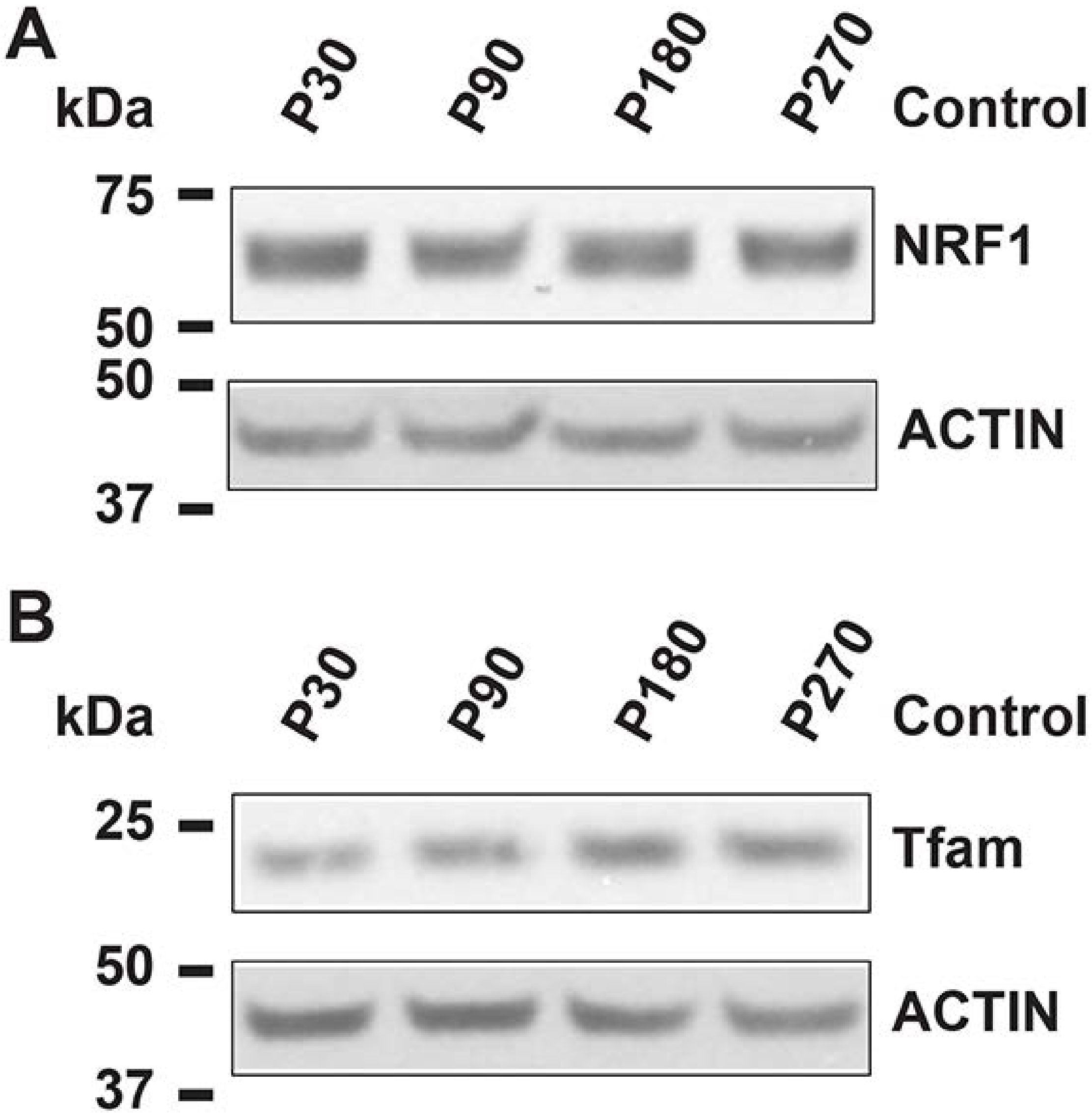

Supplemental Figure 2. PGC-1α downstream effectors NRF1 and Tfam expression in cerebellum of P30-P270 wild-type control mice. Western blotting of cerebellar homogenates (30 µg per lane) showing NRF1 (**A**) and Tfam (**B**) levels as well as actin as an internal control in the cerebellum of wild-type controls at postnatal days P30, P90, P180, and P270 (n=5-8 mice per time-point).

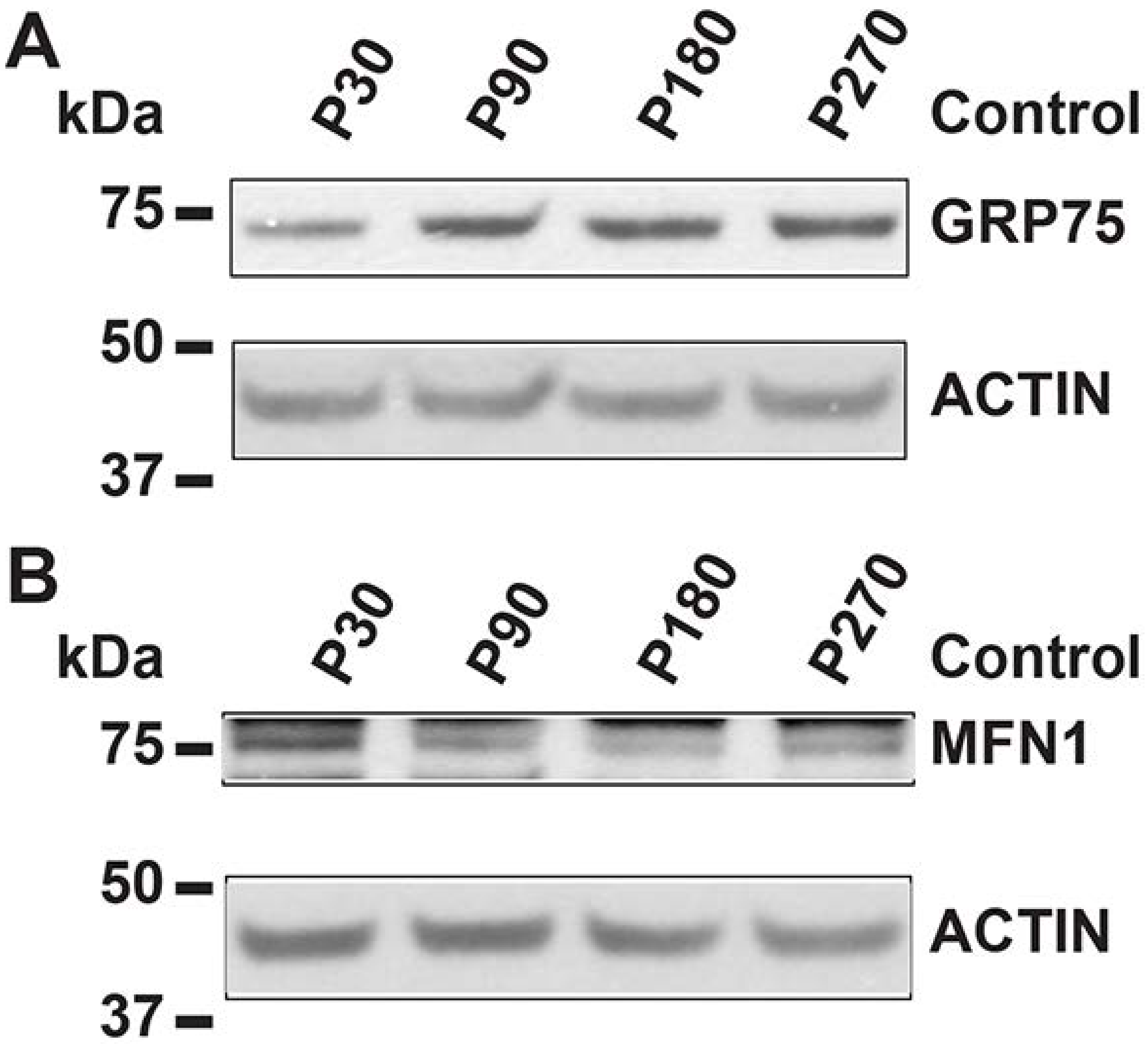

Supplemental Figure 3. Mitochondrial markers GRP75 and mitofusin-1(MFN1) expression in cerebellum of P30-P270 wild-type control mice. Western blotting of cerebellar homogenates (30 µg per lane) showing GRP75 (**A**) and MFN1 (**B**) levels as well as actin as an internal control in the cerebellum of wild-type controls at postnatal days P30, P90, P180, and P270 (n=5-8 mice per time-point).
